# The economic performance of mango integrated pest management practices at different scales of production

**DOI:** 10.3389/finsc.2023.1180568

**Published:** 2023-09-13

**Authors:** Kelvin Mulungu, Beatrice Wambui Muriithi, Menale Kassie, Fathiya Mbarak Khamis

**Affiliations:** International Centre of Insect Physiology and Ecology (ICIPE), Nairobi, Kenya

**Keywords:** fruit fly, mango, profitability, Kenya, value–cost ratio, net present value

## Abstract

Integrated pest management (IPM) strategies are being promoted to suppress tephritid fruit fly infestation and reduce economic damage in mango production. However, research on their economic performance across different mango production scales (measured by the number of mango trees) is limited. This study estimated the economic benefits of IPM practices (parasitoids, orchard sanitation, food bait, biopesticides, male annihilation technique, and their combinations) in Kenya’s small-, medium-, and large-scale mango production systems. We used the value–cost ratio (VCR) and net present value methods to estimate the heterogeneous economic performance of IPM practices using data from two unique farm surveys. On average, all IPM practices were profitable across various production scales. However, we found that these practices were more profitable for medium-scale farmers than for small- and large-scale farmers. The results show that farmers need a minimum of 9–17 trees, depending on the practice used, to break even and that there are little to no economic benefits to using IPM practices for farmers with more than 320 mango trees. The male annihilation technique was the most profitable practice, with a VCR of 36, and consequentially, the most adopted practice across all scales of production. Overall, we found significant heterogeneity in the profitability of IPM practices across different scales of production. The reason for the lack of profitability of IPM on large-scale farms remains unclear and warrants further investigation.

## 1 Introduction

In Kenya, mango production provides significant employment, with over 200,000 people involved in the value chain during each season (1). It accounts for 10% of the country’s total horticultural value and, through foreign exchange, provides more than $1.6 million annually (2). Mango is the second most important fruit in Kenya after banana (2). The main challenge faced by mango producers is fruit infestation caused by the invasive oriental fruit fly, *Bactrocera dorsalis* (Hendel) (3–5). Such infestations result in annual global mango losses of more than $1 billion in Kenya and more than $42 million in Africa (6). In Kenya, fruit flies are the cause of 40% to 80% of fruit damage, leading to a high cost of production, poor-quality fruit, high post-harvest losses, and loss of market value. In addition, the stringent trade restrictions imposed to ensure that exported mangoes are free from fruit fly infestation have limited mango producers’ competitiveness and potential earnings (7). Kibira et al. (8) estimated that approximately 57% of mangoes were rejected by buyers due to fruit fly infestation concerns.

Therefore, sustainable measures to address the threat posed by fruit flies to mangoes are needed. Although pesticides are available, their adoption by smallholder farmers is low. This is attributed to the cost and the harm they cause to the environment and human health (9). Therefore, as a means of providing a sustainable and local solution, the International Centre of Insect Physiology and Ecology (ICIPE) and its partners have developed integrated pest management (IPM) options (10). IPM is promoted over pesticide use due to its smaller negative impact on the environment, human and animal health, and production costs (11, 12). Pesticides harm the environment, and smallholder farmers often misuse them, causing health issues (13). In addition, IPM has spill-over benefits for the production of other fruits, such as avocados, bananas, and papaya (14).

However, no comprehensive analysis of the performance of fruit fly IPM practices across different production scales has been carried out (14–16). Existing studies (15, 17, 18) assume that IPM performance is homogeneous across all scales of production; however, in reality, strategies may not be appropriate for all farmers. As Grasswitz (19) argues, “In IPM—as in other areas of production—the applicability of certain strategies or tactics is scale-dependent, and what is relevant to large-scale production is not always relevant to, or appropriate for, small-scale production (and vice versa)”. In mango production, where an average tree takes 4–7 years to bear fruit, the scale of production is even more important (20, 21). Heterogeneity in returns across production scales may not benefit all farmers (22). Hence, estimating the heterogeneous impact of fruit fly IPM is crucial to promoting context-specific IPM.

However, despite these mixed results, few studies have attempted to understand if the lack of adoption of IPM by farmers with large orchards is because of the economic performance of IPM when implemented in large orchards. Whittaker et al. (23) found that the losses resulting from implementing IPM increased with an increase in farm size. In some contexts, packages of such things as pheromone dispensers are available in sizes that exceed the needs of farmers with big orchards, making them less effective (19). Mulungu et al. (24) showed that the impact of the adoption of most IPM practices was greater for those with a smaller number of mango trees in Kenya. Still in Kenya, Ronner (25) showed that mango revenue was higher for large-scale farmers, but medium-scale farmers had a higher net income (revenue less costs) per hectare. However, no comprehensive economic analysis has been carried out to understand the performance of IPM packages at different production scales.

This study estimates the economic performance of fruit fly IPM across three scales of mango production. We defined the three scales of mango production as follows: small scale (1–50 trees), medium scale (51–199 trees), and large scale (≥ 200 trees). The categorization of farmers into small-scale, medium-scale, and large-scale production follows that described in the study by Abdulai (26), who came up with these categories after consulting with experts in Kenya. This classification agrees with most definitions of small- and large-scale farmers in mango production (26, 27). The contributions of this study are twofold. First, we determine the economic return on IPM investments using the value–cost ratio (VCR) and net present value (NPV) by production scales to determine the economic return considering all costs and potential revenue. Second, we estimate the breakeven number of trees, which is the minimum number of trees required to cover all the costs of IPM and generate a positive profit level. Finally, we determine if the economic analysis agrees with farmer practices on the ground by determining if the most profitable practices are those that are most adopted.

## 2 IPM practices

The fruit fly IPM promoted by ICIPE consists of five practices. In this section, we discuss each individual component.

### 2.1 Parasitoids (P)

Parasitoids are beneficial organisms that undergo one or several of their developmental stages in their hosts, eventually killing the host upon emergence. In mango IPM, a classic biological control strategy, two exotic parasitoid species are released into mango farms. These two species are *Fopius arisanus* (Sonan), which attacks the egg stage, and *Diachasmimorpha longicaudata* (Ashmead) (Hymenoptera, Braconidae), which attacks the larval stage of the oriental fruit fly, *Bactrocera dorsalis* (28). When released into the environment, these parasitoids persistently attack the various stages of the fruit fly pest, suppressing the pest population. They are currently available for free from ICIPE, and farmers are not paying for them.

### 2.2 Orchard sanitation using the augmentorium (OS)

The augmentorium is a tent-like structure composed of resilient netting with a mesh size that confines adult flies that emerge from diseased fruit while allowing parasitoid wasps from infested fruit to flee back into the surrounding environment. It performs the dual functions of parasite conservation and orchard sanitation (29, 30). Depending on the field circumstances, the basic augmentorium costs Ksh10,000, can service 100 trees, and lasts up to 4 years on average (ICIPE staff, personal communications).

### 2.3 Biopesticide (BIOP)

The biopesticide marketed as Mazao CAMPAIGN^®^ (Real IPM Company Ltd, Thika, Kenya) is a mycoinsecticide comprising an entomopathogenic fungi (*Metarhizium anisopliae* strain ICIPE 69) that targets pupating larvae and pupal stages of the fruit fly. The conidia germinate on contact with the insect cuticle, producing a germ tube that penetrates the hemolymph where fungus proliferates, eventually killing the host. This biopesticide is applied as a soil drench underneath the tree canopy at a final rate of 200 mL per 250 trees (combined at 15 mL of product per 20 L of water) (31).

### 2.4 Male annihilation technique (MAT)

The male annihilation technique is a high-density trapping station that comprises an attract-and-kill bait for the control of *Bactrocera dorsalis* males. The bait station consists of a 5 cm × 5 cm wooden block, impregnated with a lure (methyl eugenol) and a contact insecticide (malathion). Methyl eugenol is both a sex- and species-specific male lure. It is advised that the bait station be replaced every 4 weeks and that one trap be set per 20 trees (32).

### 2.5 Food bait (DuduLure^®^) (FB)

Food bait is a component of a protein diet that is highly attractive to both male and female fruit flies. In general, fruit flies need specific proteins to survive and mature, and female fruit flies in particular need proteins for their eggs to mature. This food bait attracts all common species of fruit flies, including *Bactrocera dorsalis*, *B. curcubitae*, and *Cerratitis capitata*. The food bait can be used in either spot sprays application on the canopy or a bait trap. However, spot sprays are easier for rapid application in medium–large-scale orchards. In spot spray application, the food bait is diluted to a final a concentration of 7% with water, after which a (bio-)insecticide is added (for instance, 0.2g/L of Spinosad), after which 50 mL of the mixture is sprayed per square meter (translating into 3.5 mL per tree) using a sprayer capable of releasing 4,000 µm–6,000 µm droplets on the canopy. The sprayed area of the mango tree canopy then attracts both adult male and female fruit flies. When mango fruits reach the “golf ball stage”, application begins, and it lasts until the end of the harvest. Fruit flies consume the bait and the poison, which kills them before they can infest the fruits (10). The food bait can also be applied in a Lynfield trap and with a killing agent.

## 3 Methods

### 3.1 Data

To estimate the economic performance of the practices and their combinations, we had to obtain costs and benefits data. We used data collected in 2014 to obtain the benefits of IPM. These data were collected to determine the impact of IPM packages at the household level (15). Muriithi et al. (15) conducted a randomized controlled trial (RCT) to test the effect of different IPM practices on mango production. The main goal of this RCT was to evaluate the impact of various combinations of fruit fly IPM. Approximately 1,223 mango farmers were randomly allocated to receive different IPM practices in Meru County, one of Kenya’s largest mango-growing regions. This study estimated the effect of different practices on mangoes damaged by fruit flies, net income from sales, and reduction in expenditure on pesticides, labor costs, and revenue. We used these data to obtain the necessary parameters, such as the costs and benefits of IPM. Muriithi et al. (15) estimated the benefits of IPM but for the overall sample and not by farmer category. **Table 1** presents the distribution of farmers by production scale. Many of the farmers are small scale (approximately 60%), with large-scale farmers being the minority (approximately 8%). Upon completion of the baseline survey, the farmers were randomly allocated to different IPM practices and received a 2-day training course on the different fruit fly IPM technology practices. The first day of the training was theory based, and the second day included practical experience on a chosen farmer’s field. Further backstopping at the household level was conducted to monitor the implementation of the different IPM packages.

**Table 1:** Distribution of mango farmers by production scale.

Category	Frequency	Percentage of total (%)
Small scale (1–50 mango trees)	732	59.85
Medium scale (51–199 mango trees)	395	32.3
Large scale (> 200 mango trees)	96	7.85
Total	1,223	100

A follow-up survey targeting the same households that had been interviewed at baseline was conducted between May 2014 and June 2014, capturing information on the production season from May 2013 to April 2014. To determine the benefits (value) from each IPM, we multiplied the reduction in mangoes damaged by fruit flies (in accordance with the method described by 15) by the price of the mangoes. To obtain parameters for our economic performance analysis by production scale, we re-estimated the impact model for each farmer category, using ANCOVA to account for the small sample size (33) after we split the sample. Reduction in mango damage due to fruit flies was chosen as it is a direct measure of the impact of IPM, and this allowed us to calculate the value of each IPM practice at the prevailing prices. The practices and their combinations that we consider and the benefits for each farmer category are shown in **Table 2**, with parasitoids and orchard sanitation (POS) as the common treatments. For all practices, the impact on the reduction in mangoes damaged by fruit flies was greatest for medium-scale farmers, followed by small-scale and large-scale farmers.

**Table 2:** Benefits of different IPM practices across different scales of production.

Treatment	Benefit (reduction in mangoes damaged—kg/acre)		
	Small scale	Medium scale	Large scale
Treatment 1: POS	−931.64	−1,779.59	−1,364.25
Treatment 2: POS + male annihilation technique (MAT) (MAT)	−1,771.99	−2,113.18	−1,458.81
Treatment 3: POS + application of food bait (FB)	−1,735.21	−2,154.5	−1,651.64
Treatment 4: POS + biopesticide (BIOP)	−1,493.07	−2,017.04	−1,240.31
Treatment 5: POS + MAT + FB	−1,978.19	−2,167.14	−1,615.37
Treatment 6: POS + FB + BIOP	−1,581.64	−1,908.57	−1,571.43
Treatment 7: POS + MAT + BIOP	−1,608.1	−2,104.42	−1,651.07
Treatment 8: POS + MAT + BIOP + FB	−1,372.14	−2,076.25	−1,579.36

The numbers show the reduction in the quantity of mangoes damaged due to fruit flies in kg/acre at different production scales. Overall, we observed a huge reduction in the number of mangoes damaged due to fruit flies for medium-scale farmers, as can be seen from the above values.

For the cost data, we used the costs detailed in the study by Muriithi et al. (15) and combined these with primary data obtained from scientists and agro-dealers on the costs of the chemicals and equipment. The total cost of each IPM practice is the sum of the IPM investment and labor costs. The unit costs and application rates of IPM practices are listed in **Table 3**. For labor costs, the labor required to perform each activity was multiplied by the prevailing wage rate. The labor costs of each IPM component were negligible, except those for the augmentorium and food bait.

**Table 3:** IPM practices rates, costs, and lifespan.

IPM practice	OS	BIOP	MAT		FB
Components	Augmentorium	Biopesticides	Lynnfield trap	Methyl eugenol blocks (lure)	Protein bait (spray)
Application rate	1 per 100 trees	200mL per 250 trees	1 per 20 trees	1 per 20 trees per 1 month	3.5mL/tree
Frequency of application	Whole season*	Once per season	Whole season	Once per month	Once per week
Longevity	4 years	Every season	2 years	4 weeks	1 week
Cost per unit (Ksh)	10,000	2,000 per 200mL	80 per trap	170 per block	500 per 400mL
Cost per tree per year (Ksh)	25	8	46.6		56
Labor cost per tree per year (Ksh)	76^a^	2.4^b^	1.7^c^		47^d^
Total cost per tree per season (Ksh)	101	10.4	48.3		103

USD 1 = KSh100 at the time of the survey.

a It takes approximately 2.3 person-days to carry out orchard sanitation for an average farmer with 14 trees, with an average wage rate of Ksh225, which, when adjusted for inflation to 2021, translates to Ksh76 per tree per year.

b It takes approximately 5 minutes to spray under each tree once the solution has been mixed.

c It takes approximately 10 minutes to change the lure in the trap and clean the trap of dead fruit flies.

d It takes 5 minutes to spray each tree each week.

*Season is defined as 5 months from the flowering stage to the completion of the harvest.

The second data source used was primary data collected by Midingoyi et al. (16). These data were obtained from a random survey of 633 households from four major mango-growing counties in eastern Kenya, namely, Embu, Machakos, Makueni, and Meru. Using these data, we determined the most-adopted IPM practices using descriptive statistics. We also obtained further information from scientists involved in IPM research, such as the recommended application rates for some chemicals.

## 4 Economic analysis

Following the empirical literature, we used the VCR and NPV to evaluate the profitability of combinations of IPM (34–37). The VCR provides the ratio of the additional benefits of adopting IPM to the additional costs (i.e., the amount of money earned relative to that spent). This approach is preferred when we do not observe all other costs, which are, on average, likely to be similar across households. We defined VCR (36) using Equation (1) as follows:


(1) $$VCR=\frac{\sum_{t=0}^{n}\frac{B_{t}}{{\left(1+r\right)}^{t}}}{\sum_{t=0}^{n}\frac{C_{t}}{{\left(1+r\right)}^{t}}}$$

where *B_t_* is the additional benefits (reduction in the quantity of mangoes damaged due to fruit fly by price, assumed constant across the years) in year *t*;*C_t_* is the additional costs associated with the technology in year *t; n* is the number of years (4 years for the augmentorium); and *r* is the discount rate. Although the VCR is commonly used, some of its weaknesses include that the figures have to be converted into actual monetary value and that it does not indicate the actual costs and benefits (38). Therefore, we combined this measure with the NPV.

The VCR is calculated for all farmers owning a given number of trees and the break-even number of trees; that is, the number of trees at which VCR>1 is determined. Although a VCR of 1 is used as the break-even point to account for risk, opportunity costs, and additional labor costs, a VCR of ≥2 is considered the minimum value for farm households that show an interest in adopting a technology (34). A VCR value greater than 1 means that costs for that practice are recovered, whereas a VCR of 2 represents a 100% return on the money invested in that practice (39). For each IPM practice’s break-even number of trees, we calculated the proportion of mango farmers who could not profit from that IPM practice.

We also combined VCR with NPV analysis. The NPV represents the current value of all future cash flows (i.e., earnings minus costs) generated from IPM practices. The internal rate of return is less preferred because we do not observe all costs. The formula for the NPV is defined in Equation (2) following the method described by Mujuka et al. (36), as follows:


(2) $$NPV=\sum_{t=0}^{n}\frac{\left(B_{t}-C_{t}\right)}{{\left(1+r\right)}^{t}}$$

An NPV>0 indicates that the practice is profitable. Among the weaknesses of the NPV is that it does not provide information about the initial investment. One project could have a higher NPV in comparison with another alternative but also have a larger investment, such that the NPV-to-investment ratio is high (40). Furthermore, NPV is very dependent on the discount rate. However, in our case, the IPM packages required comparable initial investments, and we used a reliable discount rate.

All treatments involved parasitoids and orchard sanitation. Parasitoids cannot be restricted to a single farm. Orchard sanitation is also a key practice of IPM that reduces the number of fruit flies and maintains the good health of orchards—similar to weeding in crops. However, orchard sanitation is costly and labor intensive. Therefore, to determine the effects of individual IPM practices, an additive effect was assumed. We subtracted the effect of POS to obtain the effects of individual and combined practices without POS. We then determined the VCR of the individual practices and their combinations and determined the break-even number of trees.

To determine the relationship between the number of mango trees and the economic performance of IPM packages, we econometrically estimated a quadratic model of the form:


(3) $$VCR_{i}=\beta_{0}+\beta_{1}\left(num\;of\;trees_{i}\right)+\beta_{2}{\left(num\;of\;trees_{i}\right)}^{2}+\;\varepsilon_{i}$$

where *num of trees* is the number of mango trees, and *ε_i_* is the error term. From this estimation, we plotted the relationship between VCR and the number of mango trees to determine the threshold number of mango trees at which the adoption of different IPM packages is not optimal. Note that, since this is an econometric estimation, results can be inferred with higher a level of confidence than with descriptive statistics.

Finally, we used Midingoyi et al.’s (41) data to assess farmers’ most-adopted IPM practices by production scale. This random sample, collected from a different area where households were not randomly encouraged to adopt, helped us to understand which packages are common among farmers. From this analysis, we can understand if the most-adopted practices are also the most profitable.

## 5 Results and discussion

### 5.1 Economic performance of IPM practices across different scales of production

**Figure 1:**
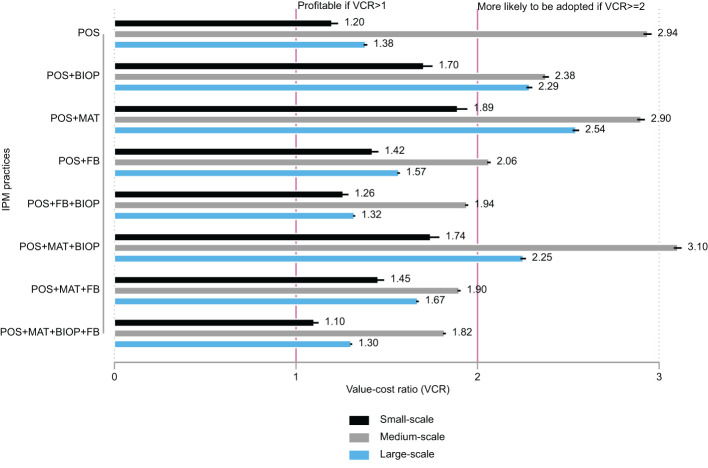
Value–cost ratios of IPM practices by production scale. The small black line at the end of each horizontal bar represents the standard error of the mean. For all practices, results show that medium-scale farmers saw the greatest benefits, as indicated by the VCR.

**Figure 1** shows the mean VCR and its standard error for each IPM practice and production scale. The VCR was calculated for each farmer using the benefit estimates obtained from the econometric results and the information on costs from **Table 1**, all adjusted for the number of trees. Because the damaged mangoes are per acre, with each acre containing 72 trees (15), we adjusted the number of trees for each production scale. We judged the profitability of IPM practices based on two rules: they were profitable if they had a VCR>1, and they were more likely to be adopted by risk-averse farmers if they had a VCR ≥ 2. The results indicate that all IPM practices used in this study were profitable (VR>1). However, all IPM practices were less likely to be adopted by risk-averse, small-scale farmers (VCR<2). IPM practices were more profitable for medium-scale farmers, with all, except for POS+FB+BIOP, POS+MAT+FB, and POS+MAT+BIOP+FB, having VCR values of more than 2. This corroborates the findings of the study by Ronner (25), namely, that medium-scale farmers had marginally higher incomes and yields from mango production. This suggests that there is an inverted-U relationship between production scale and efficiency (VCR), similar to what we found with the net income reported in **Figure 1**. This type of relationship has been found in crop enterprises between farm size and production efficiency (42, 43).

The VCR estimates are corroborated by the values of the NPVs reported in **Table 4**. The NPV was lowest for small-scale farmers and highest for large-scale farmers. Because the NPV measures the absolute sum of all future cash flows, there seems to be a linear relationship between it and the production scale or the number of trees, with large-scale farmers having larger NPVs. The profitability of IPM practices was low for small-scale farmers because all the practices include orchard sanitation, which requires the use of the costly augmentorium. This means that small-scale farmers do not use the augmentorium at capacity, which results in low profits for them.

**Table 4:** NPV for IPM practices by production scale.

IPM bundle	Small scale	Medium scale	Large scale
POS	4,804	73,330	67,235
POS+BIOP	14,301	54,952	234,025
POS+MAT	17,529	74,379	273,983
POS+FB	12,412	65,484	163,700
POS+FB+BIOP	8,626	59,858	96,797
POS+MAT+BIOP	15,349	86,548	235,236
POS+MAT+FB	13,403	56,519	197,290
POS+MAT+BIOP+FB	4,648	53,126	93,785

All figures are in Kenyan shillings (Ksh). Ksh100 = 1 USD. Large-scale farmers see the greatest benefits, as measured by the NPV, and small-scale farmers see the least benefits. However, the NPV measured absolute benefits.

In practice, farmers with few mango trees share the augmentorium. This is in contrast to our calculations, which assume that the augmentorium is used on an individual-farm basis. To determine the profitability of the fruit fly IPM when farmers share the augmentorium, we recalculated the VCR and NPV for small-scale farmers using the cost of the augmentorium shared. **Table 5** shows an example of the VCR and NPVs if seven farmers share an augmentorium. The average number of trees on small-scale farms is 14. Small-scale farmers share the cost of the augmentorium among seven households ((100/14)~7), each with an average of 14 trees. Sharing is necessary only for small-scale farmers since they use the augmentorium below capacity. All IPM practices have positive NPV and high VCR values, with the lowest being close to 2 (POS+FB+BIOP=1.92). This result shows the importance of an augmentorium being shared and used to full capacity for its cost effectiveness to small-scale farmers.

**Table 5:** VCR and NPV when seven small-scale farmers are sharing the augmentorium.

IPM bundle	VCR	NPV
POS	2.53	13,375
POS+BIOP	3.49	22,872
POS+MAT	3.89	26,101
POS+FB	2.38	20,983
POS+FB+BIOP	2.08	17,198
POS+MAT+BIOP	3.49	23,920
POS+MAT+FB	2.40	21,974
POS+MAT+BIOP+FB	1.80	13,219

All figures are in Kenyan shillings (Ksh). Ksh100 = 1 USD. POS+MAT is the most beneficial package as measured by both the VCR and the NPV. The package that combines all IPM practices is the least beneficial.

Furthermore, to confirm the existence of the inverted-U relationship suggested by the VCR results, we created a model with the VCR from each treatment as the dependent variable and the log of the number of trees and the square as the dependent variables. This quadratic model indicates the nature of the relationship between profitability and the number of trees by checking the signs of the coefficient on the level and square number of trees variable. The results in **Table 6** confirm that there is an inverted-U relationship between the number of trees and profitability.

**Table 6:** Effect of number of trees on profitability (VCR) for each IPM practice.

	(1)	(2)	(3)	(4)	(5)	(6)	(7)	(8)
VARIABLES	POS	POS+ BIOP	POS+ MAT	POS+FB	POS+FB+ BIOP	POS+MAT+ BIOP	POS+MAT+ FB	POS+ MAT+BIOP+ FB
Log (number of trees)	1.8017***	1.6684***	1.9622***	1.4391***	1.3455***	1.9996***	1.3440***	1.1310***
	(0.0861)	(0.0314)	(0.0389)	(0.0250)	(0.0284)	(0.0539)	(0.0190)	(0.0275)
[Log (number of trees)] × [Log (number of trees]	−0.1619***	−0.1647***	−0.1883***	−0.1520***	−0.1421***	−0.1873***	−0.1441***	−0.1110***
	(0.0119)	(0.0043)	(0.0054)	(0.0034)	(0.0039)	(0.0074)	(0.0026)	(0.0038)
Constant	−2.4725***	−1.7599***	−2.1916***	−1.4274***	−1.3834***	−2.3872***	−1.2228***	−1.1853***
	(0.1511)	(0.0550)	(0.0683)	(0.0439)	(0.0498)	(0.0946)	(0.0333)	(0.0483)
Observations	1,073	1,073	1,073	1,073	1,073	1,073	1,073	1,073
R-squared	0.6244	0.8876	0.8868	0.8774	0.8296	0.8213	0.9107	0.8280

Standard errors in parentheses.

*** p<0.01, ** p<0.05, * p<0.1.

For all practices, the results show that there is an inverted-U relationship between the number of mango trees and the economic performance of that practice. These results were obtained from Equation 4 using OLS.

**Figure 2:**
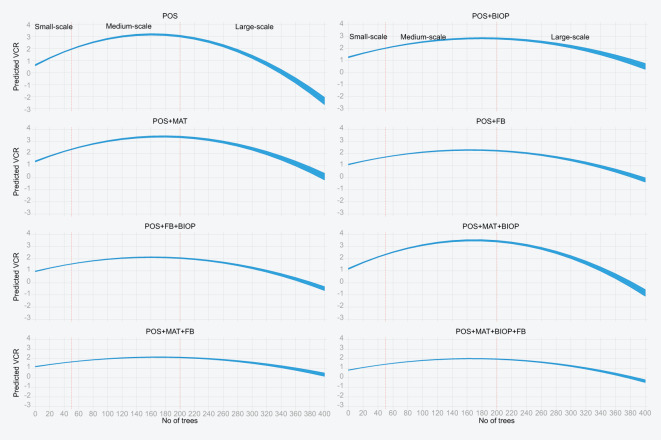
Relationship between the number of trees and profitability (VCR). The graphs have a common *x*-axis and different *y*-axis. The *y*-axis is the VCR, and the *x*-axis is the number of trees. The title of each graph is the IPM practice, the VCR for which is plotted against the number of trees. For easier plotting, the number of trees was truncated at 400 (only 39 out of 1,223 households had more than 400 trees). The orange lines in each graph mark the cutoff points for medium scale and large scale, with the resulting three sections representing the small-scale, medium-scale, and large-scale categories of farmers. A practice is not profitable if the VCR< 1. From the graph, most practices are not profitable as the number of trees exceeds approximately 320–340 trees. The plotted line is the predicted VCR, and the band around the line represents the 95% confidence interval.

In **Figure 2**, the relationship between the number of trees and profitability (VCR) is plotted with the marginal effects from **Table 6**, with the independent variable transformed back from log to number of mango trees. The visual presentation confirms that there is an inverted-U relationship between the production scale and number of trees. Profitability is initially low with a smaller number of trees (small-scale farmers) and then increases, before decreasing as the number of trees exceeds approximately 160–180 trees. Overall, IPM practices are most profitable for medium-scale farmers and the least profitable for large-scale farmers. As the number of trees approaches 320–340, most practices are no longer profitable (VCR<1). This finding is plausible given that these practices are not mechanized and are carried out using human labor. For example, it would be costly to manually spray a biopesticide onto three trees.

**Figure 3:**
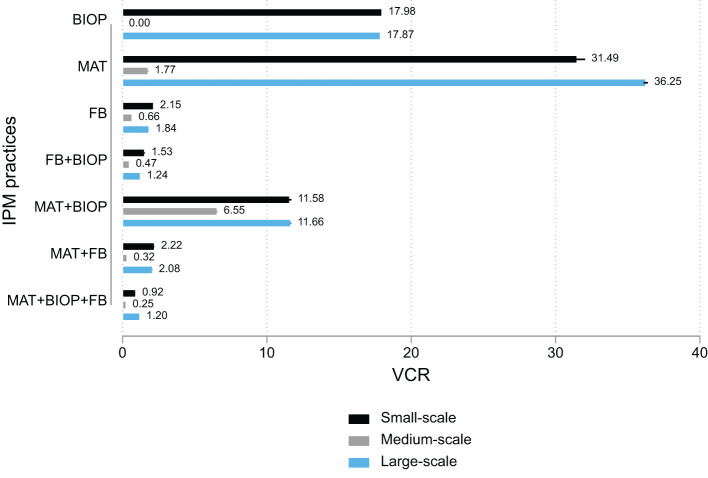
VCR for IPM practices without POS.

All IPM practices have POS in common. To understand the economic return of individual practices, we assumed an additive impact and subtracted the benefit of POS from other treatments to obtain the benefits only for other practices without POS and compute the VCR (**Figure 3**) and NPV (**Table 5**). Among small-scale farmers, individual practices on their own seem profitable, with MAT having a VCR above 35. Most fruit fly IPM practices are not profitable for medium-scale farmers, except for BIOP, FB, and their combinations. The same is true for large-scale farmers, with MAT having the highest VCR. These results show that individual technologies, that is, those without parasitoids and orchard sanitation, are useful only for small orchards. They also demonstrate that the key to successful IPM is integrating different practices to achieve synergistic benefits.

### 5.2 Break-even number of trees

The results for the break-even (VCR>1) number of trees are shown in **Table 7**. For POS and POS+MAT+BIOP+FB, 17 trees are required to break even. This is because of the high cost of purchasing an augmentorium for POS and the small benefits of using it when not combined with other practices. For POS+MAT+BIOP+FB, this is mainly because of the high cost of using all practices, which does not confer significantly greater benefits than using individual practices. Without sharing an augmentorium, approximately 19% of farmers would not break even when using these two practices. For the remaining practices, the break-even number of trees is between 9 and 14, and 7% and 15%, respectively, of farmers cannot make a profit if they do not share an augmentorium. Many farmers are not able to profit from the practices because of the high-cost implications of purchasing an augmentorium for individual farmers. If households share an augmentorium, and at least 17 trees are used for the augmentorium, all farmers can profit from IPM practices. For example, two farmers with 11 trees can share the augmentorium with 22 trees to break even under POS practice. When we remove the augmentorium, the break-even number of trees decreases significantly to between 1 and 3 trees for all practices. Given these observed differences in profitability, the next subsection shows the most adopted practices to determine whether or not farmers are adopting the most profitable practices.

**Table 7:** Break-even number of trees for the IPM practices—no sharing of augmentorium.

IPM bundle	Break-even number of trees	Percentage of farmers not making a profit
POS	17	19.41
POS+BIOP	11	10.78
POS+MAT	9	7.19
POS+FB	11	11.78
POS+FB+BIOP	14	14.65
POS+MAT+BIOP	10	8.36
POS+MAT+FB	11	10.78
POS+MAT+BIOP+FB	17	19.41

### 5.3 Are farmers adopting the most profitable fruit fly IPM practices?

We used data from the study by Midingoyi et al. (41) to determine which were the most adopted practices. These data contain information on a host of commonly used IPM practices. We mapped and created combinations that matched the fruit fly IPM practices described above and show the adoption frequency in **Table 8**. The male annihilation technique is the most-adopted technology across all production scales. This is in line with the results in **Figure 3**, which show that MAT is the most profitable technology and is more beneficial than the various combinations of two or more IPM practices. Whereas orchard sanitation is the second most-adopted IPM among small-scale farmers, MAT + OS is the second most-adopted technology among medium-scale farmers, the most adopted among large-scale farmers, and third most-adopted IPM among small-scale farmers. This is in line with the results reported in **Figure 1**, which show that POS+MAT was the most profitable among small- and large--scale farmers and the second most profitable among medium-scale farmers. The top five most-adopted IPM practices account for more than 70% of all IPM practices adopted.

**Table 8:** Most-adopted fruit fly IPM practices.

Small-scale farmers		Medium-scale farmers		Large-scale farmers	
IPM practice(s)	Percentage	IPM practice(s)	Percentage	IPM practice(s)	Percentage
Non-adopters	30.77	Non-adopters	28.27	Non-adopters	27.35
MAT	20.67	MAT	19.37	MAT+OS	19.23
OS	15.87	MAT+OS	16.75	MAT	18.8
MAT+OS	10.58	OS	10.47	OS	10.68
MAT+FB	5.77	MAT+FB	3.66	MAT+FB+OS	6.41
All combinations	83.66		78.52		82.47

Note that the OS practiced here is mostly the traditional OS that does not use the augmentorium.

## 6 Conclusion

This study examined the economic performance of mango IPM practices at different production scales. IPM practices in mango production systems can, on average, significantly reduce damage caused to mango by fruit flies at all production scales. Results from VCRs showed that the current IPM practices are generally profitable for more than 97% of farmers who have fewer than 320 trees and more than 17 trees. However, these IPM practices do not make economic sense for some large-scale farmers (i.e., those with over 320–340 trees). We also found that the break-even number of trees when the augmentorium was included in the analysis was approximately 9–17 trees, whereas it was approximately 1–3 trees when it was excluded. This impacted large-scale farmers the least. Although future studies are needed, this result suggests that it is difficult to use these practices on a large scale or that large-scale farmers might already be operating close to the production efficiency frontier. It is also possible that large-scale farmers have a challenge manually applying some of these practices to big orchards given that they are not mechanized. These results, however, can be understood in the context of the broad literature that shows that there is an inverse relationship between farm size and productivity (43). In another study on IPM, Whittaker et al. (23) found that the costs of IPM increased with farm size and profits declined.

Most literature that is focused on understanding the role of context on the adoption of IPM finds that farm or orchard size does not influence the adoption of IPM practices (44, 45) or finds a negative relationship (46, 47). In Benin, farmers who were more likely to pay to apply IPM methods against fall army worm had small-sized farms (48). We have shown here that the economic performance of IPM for large-scale farmers is much lower, and this is a potential reason for the reduced likelihood of IPM adoption that was observed for large-scale farmers.

New approaches that can be mechanized for large-scale application, such as air spraying of biopesticides, may be needed if more farmers expand their mango enterprises to include more than 320 trees. There is also a need for more research determining other factors that might contribute to the adoption of profitable fruit fly IPM practices. Future research should focus on understanding the reasons for the low profitability of fruit fly IPM practices among large-scale farmers. This is important for the sustainable use of the practices as large-scale farmers are sometimes opinion leaders in communities.

The augmentorium, which is used for orchard sanitation, is the most expensive piece of equipment, and we show that farmers need to use it at its close-to-maximum capacity of 100 trees to make a profit or, in the case of small-scale farmers, to share it among multiple households. Break-even analysis showed that POS would be economically viable if applied to farms with a minimum of 17 trees. The break-even number of trees for other practices ranged from 9 to 14. The use of IPM practices, such as POS+BIOP, POS+MAT, OS+MAT, and MAT, would provide consistent income if widely adopted.

The findings from this study should be cautiously interpreted as it has some limitations. In addition, the economic returns analysis did not use practically viable sharing patterns through distance to neighboring households because georeferenced household data were not available.

## Data availability statement

The original contributions presented in the study are included in the article/supplementary files, further inquiries can be directed to the corresponding author/s.

## Author contributions

KM: Conceptualization, methodology, writing—original draft. BM: Data collection, writing, editing. MK: Supervision, editing. FK: Research funds; editing, supervision. All authors contributed to the article and approved the submitted version.
